# Supplementary material for: Epitranscriptomics of Ischemic Heart Disease—The IHD-EPITRAN Study Design and Objectives
Source: Int J Mol Sci. 2021 Jun 21;22(12):6630. doi: 10.3390/ijms22126630 (PMC8235045; doi:10.3390/ijms22126630)
Supplement: Supplementary file 1 [file ijms-22-06630-s001.zip › Table S2_IHD-EPITRAN_IJMS_Proof.pdf]

TABLE S2 Comparison of the current techniques to map m^{6}A modification within RNA

Technique	Description	Strengths	Limitations
meRIP-seq [117–118]	Transcriptome-wide identification of those RNAs (or fragment regions) as m^{6}A-enriched, which bind with anti-m^{6}A-antibody prior PCR above that of input control	• Relatively low input material is needed<br>• RNA library preparation is straightforward	• Cannot offer single-nucleotide resolution (~200nt)<br>• Cannot identify multiple near located m^{6}A residues<br>• Increased risk for false positive peaks<br>• Cannot reliably distinguish m^{6}A from m^{6}A$_m$<br>• High level of variability between replicates [122]
LAIC-seq [123]	Transcriptome-wide identification of those RNAs (or fragment regions) as m^{6}A-enriched, which bind with anti-m^{6}A-antibody prior PCR above that of input control and at the same time are depleted from the post-IP supernatant	• Provides semi-stoichiometric information	• Time-consuming method<br>• Titration of the antibody empirically necessary<br>• Spike-in (methylated and non-methylated) controls necessary<br>• Inability to distinguish m^{6}A from m^{6}A$_m$
meRIP-qPCR [124]	Targeted quantification of the methylation level of a given region of interest relative to the input	• Provides semi-stoichiometric information at given specific m^{6}A site<br>• Straightforward protocol<br>• Low input material is needed	• Cannot identify stoichiometry of near m^{6}A sites<br>• High level of variability between replicates<br>• Spike-in (methylated and non-methylated) controls necessary
m^{6}A-CLIP/IP [125]	Transcriptome-wide single nucleotide resolution identification of m^{6}A sites after binding of the m^{6}A with an antibody crosslinking further with the mRNA via UV. Ultimate identification of bases via induced mutation and truncation profile	• High specificity due to chemical profiles<br>• Capable to detect multiple m^{6}A sites within a single transcript	• No stoichiometric information<br>• High input (mRNA) material required<br>• Time-consuming method
miCLIP [126]	Transcriptome-wide single nucleotide resolution identification of m^{6}A sites after binding of the m^{6}A with an antibody crosslinking further with the mRNA via UV. Ultimate identification of bases requires mutational signature at the site	• High specificity due to chemical profiles<br>• Capable to detect multiple m^{6}A sites within a peak or adjacent sequence<br>• Capable to differentiate m^{6}A from m^{6}A$_m$	• No stoichiometric information<br>• High input (mRNA) material required<br>• Time-consuming method
m^{6}A ELISA [127]	ELISA-based ready-to-use kit detecting the amount of m^{6}A-antibody-enriched RNA	• Relatively low input material is needed<br>• Standardized and commercial method	• Easily contaminated by the m^{6}A in other RNA species (in case of RNA species-targeted analysis)
LC-MS [128]	Digestion to single nucleotides to detect the m^{6}A by its physiochemical features via UV	• Low input material is needed<br>• Straightforward and standardized protocol	• Cannot identify the origin of m^{6}A if the sample material has multiple RNA species
SCARLET [129]	Quantitates m^{6}A in a specific site of interest via RNA H-site specific cleavage, splinted ligation, ribonuclease breakdown and chromatography	• Stoichiometric information for m^{6}A at the given single site of interest<br>• Accurate method	• Capable to measure only single site for each transcript in each run<br>• Time and sample consuming method
MazF [130]	Fluorescent-based site-specific quantitation of m^{6}As near ACA sequence via MazF cleavage	• m^{6}A stoichiometry at specific ACA contexts<br>• No antibody	• Uncapable to measure other than ACA sites
MAZTER-seq [120]	Transcriptome-wide detection of m^{6}A sites in ACA regions via ACA-specific MazF cleavge	• Widespread mapping method, no antibody<br>• Stoichiometric information at ACA sites<br>• Low rate of false-positives<br>• Relatively straightforward method	• Uncapable to measure other than ACA sites<br>• Cannot distinguish ACA sites closely adjacent with each other

Cont'd

TABLE S2 Cont'd (1)

Technique	Description	Strengths	Limitations
2D-TLC [131]	RNase T1 cleavage coupled with a 2D thin-layer chromatography to quantify m^{6}A in GAC regions; normalized to the total RNA adenosine content	• Accurate; cannot identify m^{6}A residues in AAC sites of rRNAs	• Cannot identify m^{6}As other than those preceded by guanosine (G)<br>• Radioactivity-requiring time-consuming method
m^{6}A-deoxyribo-zymes [132]	Ribozymes optimized to cleave RNA with m^{6}A in DRACH sequence	• Measurement of m^{6}A in the canonical consensus sequence	• Required designing dedicated deoxyribozyme for each site of putative interest
4SedTTP-RT [133]	RT-dependent truncation profile while 4Sed-TTP is used instead dTTP nucleotide during cDNA synthesis to pinpoint m6A sites	• Potential to identify each m^{6}A irrespective of the surrounding motif sequences<br>• No required input	• High false-positive rate (background truncation)<br>• High false-negative rate (eraser depletion necessary as a parallel control)
Tth polymerase [134]	Detection of m^{6}A residues by *Tth* DNA poly-merase with a primer extension as it prefers dTTP incorporation opposite A as compared to m^{6}A	• Relatively straightforward and validated	• Cannot identify multiple m^{6}As within a transcript<br>• Low throughput<br>• Reaction time and RNA concentration affect the dTTP incorporation efficiency opposite m^{6}A
RT-KTQ polymerase [135]	Detects m^{6}A residues by recording m^{6}A-induced increases in mis-incorporation rate by the RT-KTQ (a KlenTag DNA RT polymerase)	• Hold potential to detect every m^{6}A site at a single nucleotide resolution without sequence motif dependence<br>• Relatively simple and stoichiometric	• Poor to detect m^{6}A residues at the transcript 5'-end<br>• High rate of false-positive results
T3/T4 DNA ligase-qPCR [136]	Measurement of relative reduction in ligation efficiency of two probes designed to bind a site of interest close to m^{6}A when it is present by qPCR	• Potential to measure stoichiometry<br>• Straightforward protocol and validated	• Ligation efficiency can vary<br>• Low throughput
SELECT [121]	Measurement of relative reduction in ligation efficiency at the nick site formed by *Bst* DNA polymerase as m^{6}A is present during elongation	• Provides m^{6}A stoichiometry<br>• Simple protocol and validated for specific mRNA sites	• High rate of false-positive results<br>• Low throughput<br>• Two selective steps: 1) *Bst* and 2) ligation efficiencies
m^{6}A melting-qPCR [137]	m^{6}A is detected by assessing the melting con-ditions as DNA oligo has been allowed prior to hybridize with sample RNA with or without m^{6}A	• Relative stoichiometry at a given site<br>• Validated for some rRNA and snRNA sites	• Sensitivity is low
Nanopore [119,138]	Detection of characteristic m^{6}A and A disruptions to the basic signal induced by the flow of current from a membrane-embedded nanopores while the sample RNAs travel through	• No need for complex library preparation<br>• No shortcomings and bias from PCR<br>• Provides opportunity to assess m^{6}A in the native context of the transcript (e.g. isoform)<br>• Can measure the number m^{6}As within a transcript in single nucleotide resolution<br>• Can provide stoichiometry	• Current disruption by m^{6}A little different from A<br>• Cannot distinct m^{1}A from m^{6}A; accuracy ~90%
SMRT-seq [139]	Detects m^{6}A as altered incorporation of labelled nucleotides during cDNA synthesis	• Information relative to m^{6}A residue(s) with the transcript features (e.g. isoform)	• High base error rate<br>• Low sensitivity

Cont'd

TABLE S2 Cont'd (2)

Technique	Description	Strengths	Limitations
m^6A-SEAL-seq [140]	FTO-assisted oxidation to unstable hm^6A further stabilized via DTT-mediated thiol-addition (dm^6A) and consequently biotin tagged and IP for transcriptome-wide mapping	• No antibody needed, low input material<br>• Sensitive, specific and reliable above all other m^6A-seq methods in 8 tested samples<br>• No m^6A motif sequence dependence	• Currently detects m^6A with only ~200nt resolution<br>• Depends on multiple stepwise modifications with strict conditions
DART-seq [141]	Transcriptome-wide mapping of m^6A sites via C-U conversion in the m^6AC sequence by cytidine deaminase APOBEC1 fused with an m^6A YTH-binding domain (APOCEB1-YTH^{fusion})	• No antibody, very low input needed<br>• Potential to detect multiple m^6A sites within a single transcript	• High false positive rate (steric hindrance?) [140]<br>• Low sensitivity for low-abundance m^6A sites *in vitro*<br>• Dependent on transfection efficiency of the APOCEB1-YTH^{fusion} *in vivo*
PA-m^6A-seq [142]	Transcriptome-wide detection of m^6A sites via an anti-m^6A antibody covalent UV-crosslinking after PI with consequent fragmentation by RNase T1	• High-throughput<br>• Increased resolution to meRIP-seq (~23nt)	• Dependent on m^6A targeted antibody<br>• No single nucleotide resolution

Table modified and updated from Table 1 by Zaccara et al. [20]. Bolded horizontal lines group methods below as follows: ***1)*** *antibody-based,* ***2)*** *digestion-based,* ***3)*** *m^6A-sensing and RT-based,* ***4)*** *ligation-based,* ***5)*** *hybridization-based,* ***6)*** *direct, and* ***7)*** *additional methods not listed by Zaccara et al. [20] (antibody- and C-->U conversion-based).*

ABBREVIATIONS: ***APOBEC1***, C->U-editing enzyme APOBEC-1; ***Bst***, Bacillus stearothermophilus; ***CLIP***, UV crosslinking immunoprecipitation; ***DART***, deamination adjacent to RNA modification targets; ***dm⁶A***, N^6-dithiolsitolmethyladenosine; ***ELISA***, enzyme-linked immunosorbent assay; ***hm⁶A***, N^6-hydroxymethyladenosine; ***IP***, Immunoprecipitation; ***LAIC***, m^6A-level and isoform-characterization; ***MAZTER***, RNA digestion via m^6A sensitive RNase; ***mazF***, Endoribonuclease toxin MazF; ***meRIP***, methylated RNA immunoprecipitation; ***m⁶A-SEAL***, FTO-assisted m^6A selective chemical labeling; ***PA-m⁶A***, photocrosslinking-assisted m^6A (sequencing); ***qPCR***, quantitative polymerase chain reaction; ***RT***, reverse transcription/transcriptase; ***SELECT***, single-base elongation- and ligation-based qPCR amplification; ***SMRT***, single-molecule real-time (sequencing); ***Tth***; Thermus thermophilus; ***UV***, Ultraviolet; ***2D-TLC***, 2 dimensional thin layer chromatography; ***4SedTTP***, 4-Selenothymidine-5'-triphosphate.
