# Supplementary material for: Epitranscriptomics of Ischemic Heart Disease—The IHD-EPITRAN Study Design and Objectives
Source: Int J Mol Sci. 2021 Jun 21;22(12):6630. doi: 10.3390/ijms22126630 (PMC8235045; doi:10.3390/ijms22126630)
Supplement: Supplementary file 1 [file Table S1_IHD-EPITRAN_IJMS_Proof.pdf › Table S1_IHD-EPITRAN_IJMS_Proof.pdf]

TABLE S1 A selected summary of articles assessing the cardiovascular biomarkers measured in the IHD-EPITRAN study

Marker	Condition	Outcome	Effect	Method	N	Type	Reference
sST2	Admission sST2 in AHF	ACM (1yr)<br>CVM (1yr)<br>HF hospitalization (1yr)	**2.46** (1,80-3,37)<br>**2.29** (1,41-3,73)<br>**1.21** (0,96-1,52)	HR (CI 95%)	4 835	Meta-analysis	[99]
	Discharge sST2 in AHF	ACM (1yr)<br>CVM (1yr)<br>HF hospitalization (1yr)	**2.06** (1,37-3,11)<br>**2.20** (1,48-3,25)<br>**1.54** (1,03-2,32)				
	sST2 during HF	HF diagnosis	**8.49** (4,54-15,9)	DOR (CI 95%)	2 991	Meta-analysis	[100]
	Chronic HF	ACM<br>CVM	**1.75** (1,37-2,22)<br>**1.79** (1,22-2,63)	HR (CI 95%)	6 372<br>5 051	Meta-analysis	[101]
	sST2 ≥ median in STEMI	CVM & AHF (30d)	**3.57** (1,87-6,81)	HR (CI 95%)	1 239	Observational	[102]
	Discharge sST2 in AHF<br>Repeated sST2 in AHF	HF hospitalization & ACM<br>HF hospitalization & ACM	**1.30** (1,08-1,56)<br>**1.85** (1,02-3,33)	HR (CI 95%)	496	Observational	[103]
TMAO	TMAO in IHD	MCE (3yrs)	**1.30** (1,2-1,41)	HR (CI 95%)	4 007	Observational	[11]
	Association for IHD<br>Association for PAD<br>Association for MI<br>Association for CVD	——	**1.53** (1,36-1,73)<br>**1.71** (1,46-2,00)<br>**1.34** (1,20-1,49)<br>**1.52** (1,35-1,72)	OR (CI 95%)	1 876	Translational	[104]
	Stable IHD	ACM (5yrs)	**1.95** (1.33-2.86)	HR (CI 95%)	2 235	Observational	[105]
	Stable PAD	ACM (5yrs)	**2.06** (1.36-3.11)	HR (CI 95%)	935	Observational	
PAG	Stable IHD	MCE (3yrs)	**2.80** (2.17-3.61)	HR (CI 95%)	4 000	Translational	[12]
Copeptin	No diagnosed IHD	First incident IHD (6,5yrs)<br>CVM (6,5yrs)<br>ACM (6,5yrs)	**1.20** (1.08-1.33)<br>**1.36** (1.21-1.53)<br>**1.31** (1.21-1.41)	HR (CI 95%)	4 873	Observational	[10]
NT-proBNP	HF & 1 month follow-up	*CVM / HF hospitalization (3yrs)*<br>high → low*<br>low → high*<br>low → low*	 **0.41** (0.29-0.57)<br>**0.65** (0.44-0.97)<br>**0.35** (0.27-0.46)	HR (CI 95%)	1 923	RCT**	[106]
	5th quintile in stage A/B HF	ACM (10yrs)<br>HF hospitalization (10yrs)<br>MI (10yrs)<br>Stroke (10yrs)	**1.60** (1.10-2.32)<br>**1.51** (1.12-2.03)<br>**1.74** (1.20-2.53)<br>**2.05** (1.39-3.03)	HR (CI 95%)	1 228	Observational	[107]
	Emergency depart. dyspnea	HF diagnosis	**0.89**	AUC	205	Observational	[108]
	Dyspnea DDX	CHF<br>AHF	**23.4** (9.38-58,2)<br>**18.6** (13.0-26,7)	DOR (CI 95%)	5 092<br>1 436	Meta-analysis	[109]
hsCRP	>3mg/L vs. < 1mg/L	Incident IHD	**1.58** (1,37-1,83)	RR (CI 95%)	45 563	Meta-analysis	[110]
	≥ median (2.4mg/L)	*Incident ASCVD (18,4yrs)****<br>Triple lipid adjusted<br>Quadrupole lipid adjusted	 **1.33** (1,09-1,60)<br>**1.47** (1.18-1.85)	 HR (CI 95%)	9 748	Observational	[13]

Abbreviations: ***ACM***, All-cause mortality; ***AHF***, Acute heart failure; ***ASCVD***, atherosclerotic cardiovascular disease; ***AUC***, Area under the curve; ***CHF***, Chronic heart failure; ***CVD***; Cardiovascular disease; ***CVM***, Cardiovascular mortality; ***DDX***, Differential diagnosis; ***DOR***, Diagnostic odds ratio; ***HF***, Heart Failure; ***hsCRP***, high-sensitivity C-reactive protein; ***HR***, Hazard ratio; ***IHD***, Ischemic heart disease; ***IV***, Intravenous; ***MCE***, Major cardiovascular event; ***MI***, Myocardial infarction; ***NT-proBNP***, N-terminal pro hormone B-type natriuretic peptide; ***OR***, Odds ratio; ***PAD***, Peripheral artery disease; ***PAG,*** Phenylacetylglutamine; ***RCT***, Randomised controlled trial; ***RR***, Risk ratio; ***sST2***, soluble supressor of tumorigenicity; ***STEMI***, ST-elevation myocardial infarction; ***TMAO***, Trimethylamine *N*-oxide; *****, high > 1000 pg/mL, low < 1000pg/mL; ******, this study reported a *post hoc* analysis based on the original results from a PARADIGM-HF trial [112]; *******, this study reported a *post hoc* analysis based on the original results from a ARIC study [113].
